# Doctors’ opinions of information provided by Libyan pharmaceutical company representatives

**DOI:** 10.3402/ljm.v7i0.19708

**Published:** 2012-11-28

**Authors:** Mustafa A. Alssageer, Stefan R. Kowalski

**Affiliations:** 1 Department of Pharmacology, Faculty of Medicine, Sebha University, Sebha, Libya; 2 School of Pharmacy and Medical Sciences, University of South Australia, Adelaide, Australia

**Keywords:** pharmaceutical company representative, drug information, drug detailing

## Abstract

**Objective:**

To examine the opinions of Libyan doctors regarding the quality of drug information provided by pharmaceutical company representatives (PCRs) during detailing visits.

**Method:**

An anonymous survey was conducted among 1,000 doctors from selected institutes in Tripoli, Benghazi and Sebha. Doctors were asked questions regarding the quality of information provided during drug-detailing visits.

**Results:**

A questionnaire return rate of 61% (608 returned questionnaires out of 1,000) was achieved. The majority (*n*=463, 76%) of surveyed participants graded the quality of information provided as average. Approximately, 40% of respondents indicated that contraindications, precautions, interactions and adverse effects of products promoted by PCRs were never or rarely mentioned during promotional visits, and 65% of respondents indicated that an alternative drug to the promoted product was never or rarely mentioned by the representatives. More than 50% of respondents (*n*=310, 51%) reported that PCRs were not always able to answer all questions about their products. Only seven respondents (1%) believed that PCRs never exaggerated the uniqueness, efficacy or safety of their product. The majority of respondents (*n*=342, 56%) indicated that verbal information was not always consistent with written information provided. Seven per cent of respondents (*n*=43) admitted that they did not know whether or not the verbal information provided by PCRs was consistent with written information.

**Conclusion:**

Doctors believe that the provision of drug information by PCRs in Libya is incomplete and often exaggerated. Pharmaceutical companies should ensure that their representatives are trained to a standard to provide reliable information regarding the products they promote.

A doctor's knowledge of new therapeutic agents should be accurate and up-to-date (1). Prescribing decisions based on the information derived from objective or peer-reviewed sources may differ from that supplied by industry representatives. Companies claim that promotion provides scientific and educational information to doctors and that this knowledge will help to ensure positive health outcomes for patients (2).

Rational responsible prescribing assumes that a medical professional has balanced the positive and negative features of each product, and has considered all the alternatives. As such, the relationship between a pharmaceutical company representative (PCR) and a doctor should be based on the transfer of relevant and useful scientific information, and it has been acknowledged that PCR visits provide doctors with important information about new drugs (3–6).

The World Health Organisation (WHO) ethical criteria (7) emphasize that promotional practices must be reliable, accurate, truthful and not misleading. In many countries, it has been reported that pharmaceutical companies do not comply with these international standards. A number of studies have demonstrated that pharmaceutical promotional materials and representatives can provide low-quality (8–10), incomplete, unsubstantiated (11), vague and misleading claims (9, 12, 13).

Influencing a doctor's prescribing is not only the result of effective marketing, but is also related to the quality of the information provided and the susceptibility of the targeted recipient. Kerr et al. noted that the increase in prescribing of cyclooxeganase-2 inhibitors in Australia paralleled their promotion to medical practitioners as safer products than traditional non-steroidal anti-inflammatory drugs (14). Recently, the United States Justice Department fined the Glaxo-Smith-Kline (GSK) company $US 3 billion for unlawfully promoting unauthorized uses of paroxetine (Paxil) and bupropion (Wellbutrin), and for failing to report safety data about the diabetes drug rosiglitazone (Avandia) (15). Similarly, a study conducted in 2003 revealed that the excessive marketing and promotion of the drug Gabapentin caused an increase in the volume of the drug's prescription for unapproved uses and at unapproved doses (11).

Inappropriate marketing could possibly be worse in developing countries where there is limited regulatory infrastructure and a lack of independent information sources. The quality of information provided during drug-detailing visits has repercussions for potentially adopting or not adopting a particular drug into practice.

The information given by PCRs is largely unreported in Libya. The aim of this study was therefore to examine doctors’ opinions regarding the provision of drug information by PCRs, specifically focusing on risk information, such as side effects, contraindications and the perceived general performance of the PCR.

## Methods

This publication examines responses from a study, the first part of which has previously been published in the Libyan Journal of Medicine (16). The study employed a self-administered questionnaire that was circulated to 1,000 Libyan physicians in Tripoli, Benghazi and Sebha. Inclusion criteria, questionnaire administration, statistical analysis and other methodological aspects are as detailed in the prior publication (16).

For this report, the questionnaire (Supplemental table 1) sought the characteristics of the respondents and their practices and an assessment of the perceived quality of drug detailing by the PCRs, specifically addressing whether the information provided was convincing, balanced and consistent with written materials that they had received. The study was approved by the University of South Australia's Human Research Ethics Committee.

## Results

Of the 1,000 questionnaires circulated, 616 questionnaires were returned. Eight questionnaires had incomplete data so could not be used for the final analysis. Therefore, 608 (61%) of the questionnaires were included for analysis.

There were more male respondents (371, 61%) than female (237, 39%). The majority of respondents were from Tripoli 481 (79%). The majority of respondents (399, 66%) were in the younger age demographic (25–35 years). This was also reflected in the number of years of practice analysis where the largest demographic of respondents had between 1 and 3 years of practice (*n*=288, 47%). Almost half (274; 45%) of the respondents were general practitioners, and most were employed in the public sector (512, 84%) (Table 1).

**Table 1:** The demographics of the study subjects

Age	*N*	%
25–35	399	66
36–45	123	20
46–55	64	11
56–65	22	3
Gender		
Male	371	61
Female	237	39
Years of practice		
1–3	288	47
4–6	82	14
7–9	45	8
> 10	193	32
Practice setting		
Public	512	84
Private	34	6
Both	62	10
Locations of practice setting		
Tripoli	481	79
Benghazi	77	13
Sebha	50	8
Area of practice		
Residents	41	7
Anaesthesiologists	61	10
General practitioners	274	45
Surgeon	99	16
Other	42	7
Physician specialists	91	15

**Fig. 1:**
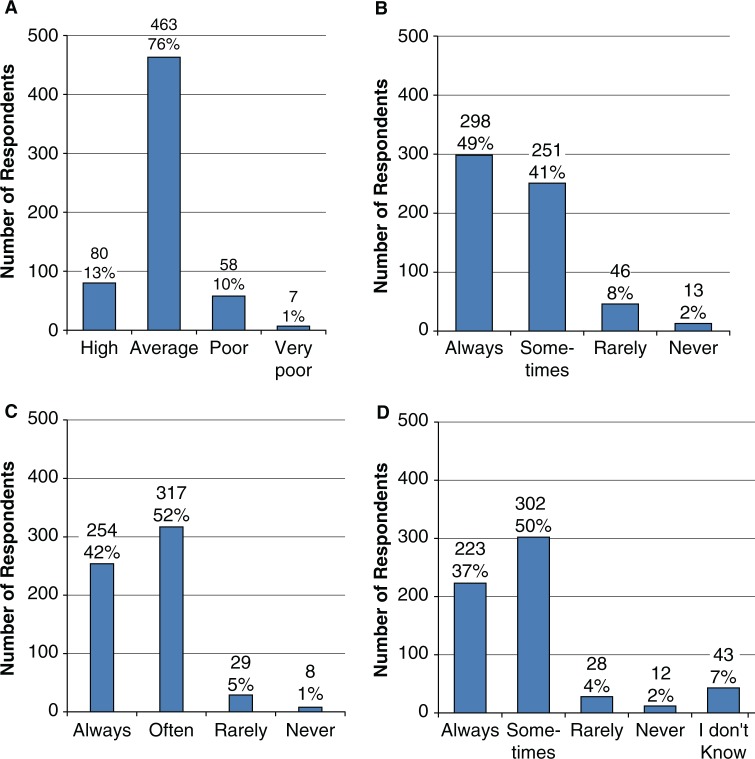
Participating physicians’ evaluation of PCRs and the quality of the information they provide. A. Grading of the quality of PCR information. B. Willingness of PCRs to answer any question about products? C. Do you believe that pharmaceutical representatives greatly exaggerate the uniqueness, efficacy or safety of their products? D. Was the verbal information consistent with the written information provided?

Only 13% (*n*=80) of respondents graded the quality of information provided by PCRs as high. The majority (*n*=463, 76%) of surveyed participants graded the quality of information provided as average and 11% (*n*=65) of prescribers graded the information provided as poor/very poor (Fig. 1A).

Over 40% of respondents indicated that contraindications, precautions, interactions and adverse effects of promoted products of PCRs were never or rarely mentioned during promotional visits (Table 2). Only 20% of respondents reported that the above information was always presented during promotional visits. The majority of doctors (65%, *n*=403) reported that alternative drugs for treatment were never mentioned by PCRs.

**Table 2:** Perceived frequency of the spontaneous provision of prescribing information for a promoted product. ‘Do PCRs spontaneously highlight the following drug information points regarding their products during visits?’ (Number, %)

	Never	Rarely	Sometimes	Always	I don't know
Contraindications	88 (14)	193 (32)	166 (27)	121 (20)	40 (7)
Precautions	69 (11)	188 (31)	188 (31)	121 (20)	42 (7)
Interactions	110 (18)	176 (29)	163 (27)	11 (18)	48 (8)
Adverse effects	97 (16)	188 (31)	156 (26)	116 (19)	51 (8)
Alternative drugs	234 (39)	169 (26)	86 (14)	68 (11)	61 (10)

Nearly half of the respondents (*n*=298, 49%) indicated that PCRs were always willing to answer questions regarding their products, but of more concern was the finding that 10% (*n*=59) of respondents believed that PCRs were rarely or never able to answer questions about their products (Fig. 1B).

Only 6% (*n*=37) of respondents indicated that information received from PCRs was never (*n*=8) or rarely (*n*=29) exaggerated. By contrast, 94% (571) of medical practitioners believed that PCRs always or sometimes exaggerated the uniqueness, efficacy or safety of their products (Fig. 1C).

Over half of the respondents (*n*=342; 56%) indicated that the verbal information was not always (i.e. never, rarely, or sometimes) consistent with the written information delivered during promotional visits. Only 37% of prescribers (*n*=223) indicated that the verbal information was always consistent with the printed materials provided. Of concern, 7% of participants (*n*=43) did not know whether or not consistent information had been provided during their encounters with PCRs. Two per cent of respondents (*n*=12) indicated that the verbal information always conflicted with the printed materials (Fig. 1D).

## Discussion

The provision of complete and balanced drug information is necessary for rational drug use. Both scientific and commercial information sources can provide doctors with the necessary information to make informed prescribing decisions. It is important, however, that the information provided by PCRs is accurate, complete and balanced. The current study found that only 13% (*n*=80) of the respondents graded PCRs’ information as ‘high quality’. The majority of the doctors (76%) graded the information provided during visits as ‘average’. PCRs often failed to mention important safety information about their products and 41–65% of the medical practitioners surveyed reported that PCRs rarely or never mentioned safety information. This result is in concordance with a number of previous studies, which reported that pharmaceutical promotional materials and representatives provide low-quality information (1, 3, 8, 9). A study performed in Sudan found that approximately one-third of 160 PCRs interviewed admitted they did not always mention contraindications, precautions or drug interactions, and only 4.3% mentioned the side effects of their promoted products during drug-detailing visits (17). It is assumed that marketers will attempt to present the positive aspects and advantages of their products, but downplay any negative information. However, by not presenting this information the credibility of the information provided is diminished and may also negatively influence the perceived truthfulness of their presentations. In other words this strategy may not be effective from a marketing perspective if it leads to the source becoming untrustworthy.

The consequence of omitting some risk-related information can also have potentially tragic consequences for patients. Medical practitioners may believe that all significant risk-related information has been presented, especially if some risks are highlighted. It may result in doctors failing to inform their patients of important considerations, and, at worst, doctors may make inappropriate prescription decisions.

Over half of the respondents (56%) indicated that the verbal information was not always consistent with the written information that was delivered during PCR promotional visits. This has also been reported in studies conducted in India where PCRs admitted that there were often inconsistencies between what they had been told to tell the doctor, what was written in flip charts used and what was in the medical literature (18).

The depth and quality of the education and the preparation of PCRs varies between pharmaceutical companies. In this study, half of the respondents (*n*=310; 51%) did not believe that PCRs were always confident about their products, and 10% (*n*=59) reported that PCRs were rarely confident during their detailing visits. Parker and Pettijohn found that doctors were not satisfied about the information they received about drugs from the PCRs, nor did they find it adequate. Subsequently, it did not give them the confidence to prescribe certain specific pharmaceutical products (19). A longitudinal survey of French physicians reported that PCRs were not fully compliant with the agreed codes of conduct, and over the years 1991–1998, an average of 27% (± 1.6%) of doctors reported that PCRs spontaneously mentioned side effects during their detailing visits. In addition, only an average of 26% (± 2.8%) of respondents found PCRs convincing (20).

PCRs should receive medical and technical training sufficient enough to enable them to provide medical and technical details in an accurate, responsible and evidence-based manner when presenting advertisements either verbally or in writing (21).

The quality of information provided during a visit is only one component of effective marketing. How the message is communicated is also important (22). PCRs promote their products by using a unique brand identity to present their products as clearly different from the competitor products. Strang et al. found that 80% of Canadian doctors believed that PCRs exaggerated their products’ effectiveness (23), while Hemminki reported that PCRs always presented their own products as the drugs of choice (11). An exaggeration of the uniqueness, efficacy or safety of pharmaceutical products was also reported in our study by 99% (*n*=602) of respondents.

Regardless of the quality of drug detailing, information provided by PCRs is designed to be consistent with commercial objectives. From an industry perspective, PCRs need be able to effectively present and market their product information (24). One company estimated that to recruit, train and support their PCRs it spent approximately $100,000 annually per representative (25). The success of a drug detailer is not assessed by the quality of information provided to the practitioners, but principally only in terms of prescription numbers and sales returns (26). Consequently, the quality of information can be compromised as it is only a secondary consideration for many PCRs (27). Idris et al. reported that 82% of PCRs admitted that they would continue to ask doctors to prescribe their product even when the competitor's product was superior to theirs (17).

From this study, it cannot be determined why some PCRs offer low-quality information. While PCRs usually possess extensive skills in sales and marketing, they should also be proficient with the pharmacotherapy required to sell their product. PCRs should also have an understanding of the codes of conduct and the ethical requirements for their industry. In a study from Yemen, 14 PCRs from both multinational and generic medicine suppliers were interviewed. The study found that the majority of PCRs were unaware of any code of conduct, and that they manipulated their promotional tools according to the marketing strategies of their companies and doctors’ engagement receptivity (28). In the Sudanese study, Idris et al. found that 74% of PCRs indicated that the training they received from their employers was inadequate (17).

The effectiveness of drug detailing is directly dependent on the quality of drug information provided, and this will have a direct effect, positively or negatively, on rational drug prescribing. If there are concerns regarding false or misleading information from inappropriately trained PCRs, accreditation or certification may become desirable or even necessary, to ensure that the prescribing patterns of physicians are not negatively affected. Lagace et al. indicated that physicians could gain positive impressions when PCRs behaved ethically and when they showed expert knowledge (29). Andaleeb & Tallman suggested that PCRs need to build and maintain long-term relationships with prescribers by providing reliable, relevant and usable information for medical practitioners (27).

Ethical issues arise if PCRs attempt to deceive medical practitioners by omitting risk-related information or by using a language that is confusing. Misleading and inaccurate drug-related information is likely to be worse in countries where independent sources of drug information are absent or restricted. In many developing countries, there is limited access to regular, up-to-date independent drug information, and medical practitioners are therefore more likely to be reliant on commercial sources of information (30, 31). Relying on low-quality information can negatively influence medicine usage, affect prescribing habits and reduce cost-effectiveness.

In Libya, advertising of any pharmaceutical preparation by words or phrases that cannot be proven is prohibited, and no manner of drug promotion may be practised which may be contrary to ethics (Libyan Health Law Act Number 106 of 1973 and its explanatory notes of 1975) (32). However, in developing countries such as Libya, the regulatory infrastructure is not well developed, and there is no effective mechanism to monitor pharmaceutical promotional activities. Therefore, in the absence of effective regulations and standards for provision of pharmaceutical promotional information, physicians should be aware of the risks of using non-independent information sources.

There are limitations to this study. The aim of this study was not to directly and objectively assess the accuracy and efficacy of the information provided by PCRs, but to question prescribers regarding their perceptions of the information provided. Further studies that directly access the quality of the information provided during visits should be performed.

## Conclusion

The survey revealed that doctors largely believe that the provision of drug information by PCRs is sometimes incomplete and biased. Incomplete presentation of risk-related information, exaggeration of product claims and ignorance of equivalent or alternative products was common. Pharmaceutical companies should ensure that their representatives have total familiarity with their products and have the skills necessary to provide high-quality drug-detailing information in accordance with the accepted codes of practice.

Establishing ethical guidelines and codes of conduct will be the first step in Libya to ensure effective and informative pharmaceutical promotion.

## Key messages

- Pharmaceutical company representatives’ information is not always accurate or unbiased.
- Risk information is often omitted or overlooked during promotional visits.
- Pharmaceutical companies should train representatives to appropriate standards.
- A code of conduct should be established for pharmaceutical promotion in Libya.
